# Prospects for the Development of the Demand for Carp in Poland among Young Consumers

**DOI:** 10.3390/ijerph19073831

**Published:** 2022-03-23

**Authors:** Magdalena Raftowicz

**Affiliations:** Department of Applied Economics, Faculty of Life Sciences and Technology, Wrocław University of Environmental and Life Sciences, 50-375 Wrocław, Poland; magdalena.raftowicz@upwr.edu.pl

**Keywords:** aquaculture, food heritage, carp, consumer preferences of Young Adult Populations, marketing place

## Abstract

Carp fishing economy in Poland has a centuries-old tradition. However, in the last decade, as a result of changes in global market trends, this industry has experienced stagnation. Still, the elimination of this niche industry may have painful consequences for the entire ecosystem and biodiversity. Hence, every effort should be made to protect and maintain the status quo. The aim of the article is an attempt to show that the development prospects for the carp market in Poland are limited, especially in the face of little interest in carp consumption by young adult consumers, who will create the demand for carp in the near future. The remedy may be to change the image of the carp together with a territorial marketing strategy that would be consistent with the preferences of the young generation. The research was conducted on the basis of a critical analysis of the literature of the subject, focus studies, questionnaires and a case study.

## 1. Introduction

The carp economy in Poland has a tradition that goes back over eight centuries. It is assumed that knowledge about carp breeding was transferred to Poland by religious orders, and later by the secular clergy (bishoprics). The oldest order that was the first to raise fish were the Cistercians, brought to Poland in the 12th century from Belgium and France. As the Christian religion strengthened, there was a need to respect numerous church fasts, the number of which exceeded 200 days a year [1], thus increasing the national demand for fish. The turn of the 16th and 17th centuries is often considered the ‘golden age of Polish pond carp fishing’. This thesis is exemplified by the fact that at that time, ponds with a unit area of 100 to over 1000 ha were built. After the Second World War, as a result of the state border change, the area of ponds in Poland decreased by over 22 thousand ha and amounted to a total of 66,525 ha, of which, until 1989 (i.e., until the political transformation in Poland), about 75% of the pond area was used by the State Fisheries Farms. The beginning of the 1990s, which initiated the functioning of the free market economy in Poland, was characterized by the appearance of completely new, private carp breeders (currently, there are about 300 carp farms in Poland) [2], who, however, still cultivated breeding traditions and the traditional form of selling carp in the form of live fish. The implementation of such sales strategies a decade ago strengthened the eating habits of Poles even more. Currently, 80–90% of carp consumption in Poland takes place around Christmas, which is closely related to Polish tradition and Christian culture, where carp is a traditional dish on the Christmas Eve table.

The aim of the article is an attempt to show that the development prospects for the carp market in Poland are limited due to the decline in domestic demand and the reluctance of the young generation to consume carp all year round. However, the elimination of this niche industry may have painful consequences for the entire ecosystem and its biodiversity. Hence, every effort should be made to protect and maintain the status quo.

Such research assumption established also the purpose of the study, which consisted in identifying consumer trends from the perspective of young consumers and changes taking place on the carp market in Poland from the carp producers’ point of view.

The research was conducted on the basis of a critical analysis of the literature of the subject, focus studies, questionnaires and a case study.

The practical aspect of the issues discussed in the article will be the possibility of using the research results by the aquaculture advisory services, NGOs, local governments and carp farms themselves interested in developing and selling their products in the future.

The structure of the article is divided into seven parts. The first part is the introduction to the carp economy in Poland. The second part introduces the importance of carp in the food economy, with particular emphasis on its non-production aspects, especially those social and environmental. The third part presents new consumer trends in the agri-food sector. The fourth part illustrates the adopted research methodology. The fifth part presents the results of the focus studies and questionnaire research conducted in the chosen target selection. Parts 6 and 7 close the considerations, presenting the discussion and conclusions in turn.

## 2. The Importance of Carp in the Food Economy

**Figure 1:**
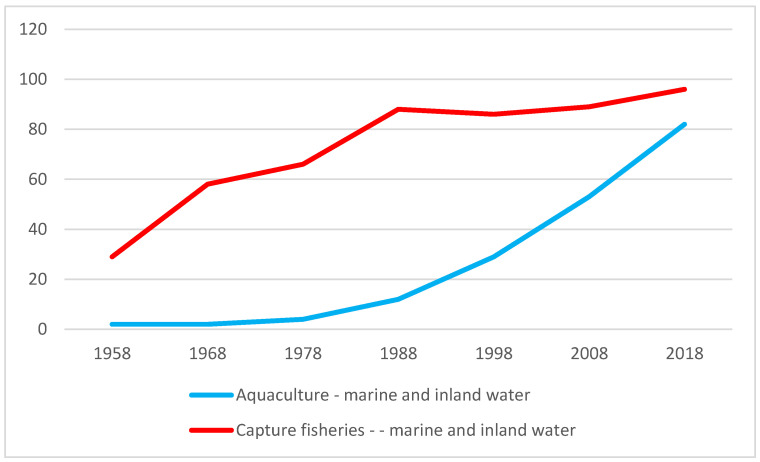
Capture fisheries and aquaculture production (in million tonnes). Source: own study based on [4].

Fish and seafood account for approx. 7% of the global food market [3]. Even at the end of the 20th century, the quantity of fish supplied for consumption was determined by catches in the seas and oceans. However, over the past two decades, there has been a significant decline in the catch dynamics of sea fish due to severe overfishing. Only thanks to the intensive aquaculture development, despite the decline in the state of sea resources, world fish production continues to show an upward trend, as illustrated in Figure 1.

Aquaculture means the breeding or farming of aquatic organisms by means of specifically developed techniques with the aim of increasing production beyond the natural environmental capacity, while the organisms remain the property of a natural or legal person throughout the breeding and farming period, up to and including the catch [5]. It covers both fish and seafood (crustaceans, mollusca and seaweed). Production takes place in ponds, pools and fairways, partitions and wells, cages, recirculation systems and other devices not mentioned above. Currently, aquaculture covers about 580 species of aquatic animals and plants [6]. World aquaculture production is dominated by China, as shown in Table 1.

According to FAO estimates, by 2030, the global share of aquaculture in relation to traditional fish catches will increase from 46% to 57% [8]. Aquaculture allows for the supply of natural animal protein and thus contributes to strengthening global food security. Statistics of using feed and water in breeding aquaculture products are also favorable in comparison to the to beef, pork or poultry, as shown in Table 2.

Table 2 shows that the production of 1 kg of beef requires 8 kg of feed and 14.5 thousand liters of water. For 1 kg of pork, 3 kg of feed is needed and nearly 6 thousand liters of water, while salmon farming seems to be the most effective and ecological because it requires only 1.1–1.2 kg of feed and 1500 L of water.

The importance of the aquaculture development is evidenced by the fact that the catch of cod (Poles’ favorite fish [11]) in the Baltic Sea has fallen from 50 thousand tones in 2015 to 21.6 thousand in 2018 [12]. Thus, the monthly consumption of fish and seafood in Poland decreased from 0.45 kg per person in 2010 to 0.27 kg per person in 2020 (i.e., by as much as 40% over a decade) [13].

In the world, the most popular farmed species of aquaculture are those belonging to the carp family: grass carp, silver carp and common carp, as shown in Table 3. 

Common carp is the third most frequently produced fish species in the world, with 97.3% of its production taking place in aquaculture [14]. Global common carp production is dominated by China. In the EU, the leader in the production of carp is Poland with an annual production of approx. 20 tons, almost entirely allocated to the domestic market (approx. 96–97%), as shown in Table 4.

For comparison, nearly 67 thousand tons of carp [16] are produced in the whole European Union. However, the consumption of carp in Poland is gradually decreasing; currently, it amounts to 0.56 kg per year/person [17]. The research has shown that it is not price that is the main obstacle to consumption, as the demand for carp is rigid. This means that lowering the price will not reflect in an increase in carp demand [18].

The non-production aspect of the carp economy in Poland also plays an important role. Social and environmental values are of particular importance here.

When it comes to the social sphere, there is a long list of benefits that local residents derive from the conducted fishing activity. In addition to traditional values such as food supply and employment guarantee, the development of fishing, processing and recreation (e.g., hotel industry, gastronomy or agritourism) may be observed, which is related to the possibility of the multifunctional development of rural areas.

When it comes to nature, research has shown that carp is a very environmentally friendly system of fish farming [19]. Moreover, carp management is one of the aquaculture systems most preferably accepted by consumers, together with their expectations for sustainable fish farming [20]. Especially the management of water resources is of great importance here. The ponds act as retention reservoirs and thus maintain a higher groundwater level. Water flow is used only in emergency situations, when the welfare of the stock is threatened as a result of e.g., high temperatures. During the season, the water is replenished only in situations of its visible losses due to evaporation or leakage through dikes. Due to the water retention system used in carp farming, these ponds have a very positive effect on the water quality in the catchment area. This contributes to the retention of very large number of factors responsible for water eutrophication during the production cycle: both nitrogen and phosphorus. The pond management also positively influences the amount of water flow in the watercourse. The research results have shown that the lower the ratio of the catchment area to the area of ponds, the more favorable this effect is [1]. Carp ponds also function as areas of rich natural value. First of all, they are a refuge for wetland birds. The extensive carp ponds serve as wetlands and provide high biodiversity habitats for protected species of birds, amphibians, reptiles, mammals and insects. Moreover, carp farms have a positive effect on the microclimate of the surrounding areas and have a much higher agro-ecological value than arable lands and grasslands. An example is the area of the Barycz Valley, where the largest carp breeding center in Europe is located (the Milicz Ponds). This area covers almost one-fifth of the total usable area of breeding ponds in Poland, while the area of land under the waters of the Barycz Valley is as much as 11%. The Barycz Valley has been included in the Ramsar Convention on Wetlands of international importance, especially as a habitat for waterfowl due to the presence of numerous ponds, wetlands, meadows and forests, which affects the development of biodiversity [21]. In the Barycz Valley, there are also three areas covered by the Natura 2000 program as areas of special protection for birds (especially white-tailed eagles, cormorants, black storks, cranes and herons (white and gray)). These are Ostoja nad Baryczą (82,026.4 ha), the Barycz Valley (55,516.8 ha) and the Lower Barycz Valley (3165.8 ha) [22]. It also houses the largest ornithological reserve, the Milicz Ponds in Poland, and the largest Landscape Park in Poland, the Barycz Valley. These areas, as one of four in Poland, have also been classified as environmentally sensitive areas, which enables the implementation of EU agri-environmental programs. Due to the unique natural and landscape values of the Barycz Valley Landscape Park, this area is an example of the need to harmonise environmental, social, economic and spatial aspects [23].

## 3. New Consumer Trends in the Agri-Food Sector

The changes taking place in the contemporary world as a result of the globalization and internationalization processes of the world economy have a significant impact on the modern consumers attitudes, especially the young ones. The change in consumer behaviour is caused, among others, by the awareness of the sustainable development principles, circular economy or the need to shorten the food supply chains [24]. There is also growing awareness of changes in local food production markets and the importance of the consumption of healthy, high-quality products, including local [25], regional [26], traditional [27] and natural [28] products, which is in line with the development of slow food [29,30], culture and food safety [31].

These trends are also present in Poland, where, after 1989, there has been a significant change in the approach to consumption [32]. Back in the 1990s, Polish consumers focused mainly on satisfying basic nutritional needs, which was consistent with the ‘food consumption model’, while in the following years, along with the economic growth and income growth, the consumption structure of Poles assumed the nature of an ‘industrial model’. The beginning of the 21st century can be, quoting [33], called ‘consumer capitalism’ characterized by a rapid increase in consumption and a significant improvement in the living standard of the societies of middle and highly developed countries, including Poland. It was then that a peculiar McDonaldization of consumption patterns took place, consisting in the massification and unification of Western values. However, in opposition to strong Western consumerism, a model of sustainable consumption has emerged, which is based on ethnocentric, environmentally friendly patterns. This model is also related to the servicisation of consumption, in which the communication process (especially in social media) and an attractive way of spending free time are of great importance (the traditional division into work and free time is disappearing).

A new consumer trend is also prosumption [34], defined as the phenomenon of intertwining consumption and production processes, which leads to blurring the boundaries between them, where consumers become producers [35] at the same time. In other words, prosumption is the expression of consumer opposition to mass, unified and standardized production. By engaging in the production process, the consumer is able to produce the final product in line with their expectations through independent design and reconfiguration. In practice, a prosumer is a consumer who meets at least two of the three conditions [36]:

1. Becomes acquainted with the opinions of other Internet users and most often personally looks for them when planning to purchase a product;
2. Describes products online or asks questions about them;
3. Participates in promotions and co-creates products, slogans or advertising campaigns.

There is also a slow change in consumer orientation on the food market from a quantitative to a qualitative approach, which is exemplified by the diminishing role of price in making decisions about food purchases [18]. In recent years, there has also been a dynamic increase in the number of Polish consumers who are looking for high-quality products with high nutritional value, produced using methods consistent with the idea of sustainable development, including organic food, produced locally and sold in short supply chains [37].

## 4. Materials and Methods

**Figure 2:**
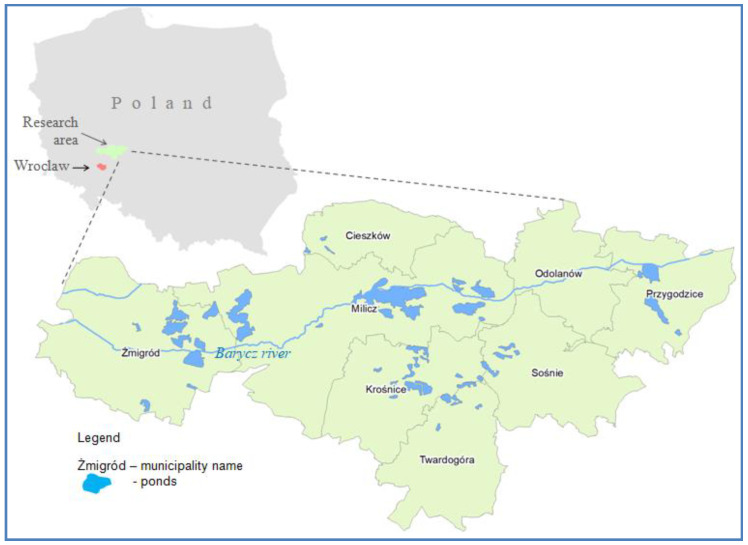
Barycz Valley. Source: own elaboration.

The research area was narrowed down to the Barycz Valley in Poland, where the largest carp breeding center in Poland and Europe is located, as shown in Table 5 and Figure 2.

The Milicz Carp from the Barycz Valley has been entered on the List of Traditional Products of the Minister of Agriculture and Rural Development [39]. It has become a key image element of the economic, natural and cultural offer in the Barycz Valley.

The first element of the research was to conduct focus studies (10 participants) combined with in-depth expert interviews with three owners of fish farms on the specifics of the carp market and the changes that producers have observed on the market. These studies were conducted in October 2019. A focus group is a qualitative research method that brings together a small group of people to answer questions in a moderating setting. The method aims to obtain data from a purposely selected group of individuals [40].

The focus study, through direct contact with producers enables the acquisition of hard-to-reach data and information. In addition, it allows for a better understanding and grasping of changes and challenges in production. In the literature, focus studies in the carp sector in Barycz Valley were already conducted in 2016 by [41].

The second element of the research was an electronic questionnaire addressed to the target group of people aged 18 to 26, which belong to the Generation Z (people born in the period of 1997–2012). The questionnaire (see Appendix A) was disseminated among students of the University of Life Sciences in Wrocław (60 km from the Barycz Valley) by e-mail and using social networks in May 2021. The selection of the sample was deliberate, and the results presented here concern 169 people. The majority of the sample was female (79%), which is correlated with the global trend, because 80% of purchasing decisions are made by women [42].

## 5. Results

Focus studies have shown that carp producers are aware of the changes taking place on the market. In their opinion, contemporary retail consumers of carp (especially young people) value a healthy lifestyle combined with healthy eating and outdoor activities (bicycles, kayaks) combined with self-realization and passion (fishing, nature walks). The aesthetics of the place and the locality of production are also important for consumers. They put emphasis on ecology combined with convenience and comfort (e.g., the possibility of making cashless transactions).

Hence, focus studies have shown that the era of live carp wholesale is about to end forever in favour of shortening food supply chains and diversifying activities. Traditional fishing farms (in a three-year production cycle with the avarage cost of production about 8–10 PLN/kg), which, until now, were mainly involved in the pre-Christmas sale of live fish, are currently obliged to expand the range of activities with new elements, as shown in Table 6.

The results have shown that diversification for fishing farms often means investing additional funds (usually EU funds) for the construction of restaurants, processing plants, smokehouses or mobile fish sales points, which leads to an increase in the number of employees, taking up new managerial skills and implementing new organizational and technological solutions. An important element of diversification is also the use of modern communication tools with the client through social media (e.g., Facebook, Google) and care for the company’s image. This raises the need to create completely new B2C relationships, in which the manufacturer must take care of partner relationships with customers and provide information about the origin of products, their history and quality. In particular, the traditional generation of Poles with carp swimming in the tub before Christmas, as a result of the natural aging process of the society, is irretrievably gone. That is why it was so important in the research to capture the perception of carp by Generation Z, who will shape the demand for carp for the next decades.

**Figure 3:**
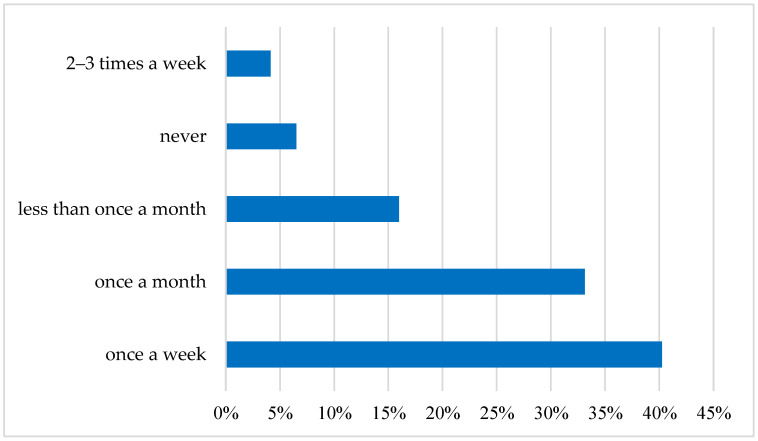
Frequency of fish consumption in Poland among the Young Adult Populations. Source: own elaboration based on data of [43].

The results of research among young Polish consumers have shown that fish are present on their menu: 40% of the surveyed consumers declared that they eat fish once a week and 33% once a month, which means that the fish diet is widely accepted by young consumers, as shown in Figure 3.

**Figure 4:**
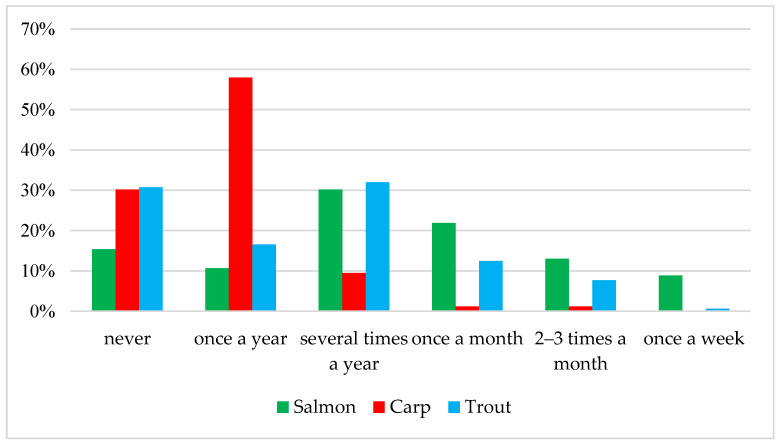
Frequency of salmon, carp and trout consumption in Poland among the Young Adult Populations. Source: own elaboration based on data of [43].

It can also be concluded that young consumers are aware of the benefits of eating fish, even though some of the respondents do not consume it because of their vegetarian or vegan diet. As for the fish species most eaten by young consumers, studies have shown that the most consumed fish is salmon (at least once a week—9%; once a month—22%). The results of the research clearly show that carp is a fish that is eaten once a year (58% of responses) or never 30%, as shown in Figure 4.

**Figure 5:**
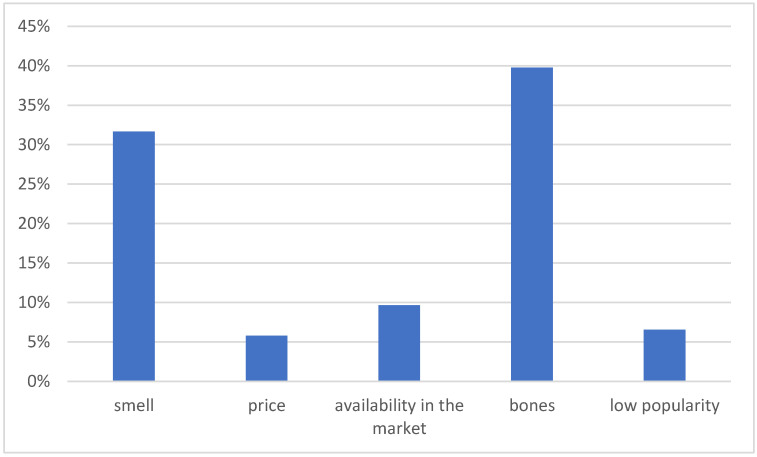
Factors discouraging the consumption of carp among the Young Adult Populations. Source: own elaboration based on data of [43].

Focusing strictly on the consumption of carp, the research has shown that the Christmas tradition was a key factor motivating the consumption of this fish for over 60% of consumers. The second of the most encouraging factors turned out to be taste, which accounted for 18% of all the answers provided. For comparison, the questions were also asked about the factors discouraging the consumption of this fish. Research has shown that the number of fish bones in the meat is the most frequently reported deterrent to eating carp. As many as 40% of respondents have a problem with the comfortable preparation or consumption of this fish because of bones. For 32% of consumers, the smell of silt is discouraging, and 10% of respondents indicated the lack of availability of carp in stores, as shown in Figure 5.

In fact, during the year, apart from the Christmas season, there is no sale of carp in popular stores and discount retailers, and it is almost exclusively possible to buy it directly from the farm. As for the price, only 6% of interviewees indicated this factor as a disincentive to consumption. Due to the fact that for many years carp was a fish prepared exclusively at home, the respondents were not inclined to order carp in a restaurant (as much as 71%). Only 14% of the respondents are willing to consider the choice of a carp dish, and 13% admit that they would be willing to order it. The price was another studied factor that could affect the attitude of young adult consumers to carp. It was assumed that the affordable price could have an incentive effect on potential carp buyers and contribute to the popularization of the carp meat consumption. The research has shown that the price would not affect their attitude to the purchase or consumption of carp, which was declared by as many as 64% of the respondents. According to the preferences of consumers, the most affordable price range per kilogram of fish in the form of a fillet is from PLN 20 to PLN 25. This option was chosen by 41% of respondents; 31% of consumers would prefer to pay less than PLN 20 per kilogram of fish fillet—this option is one of the most economical and affordable.

**Figure 6:**
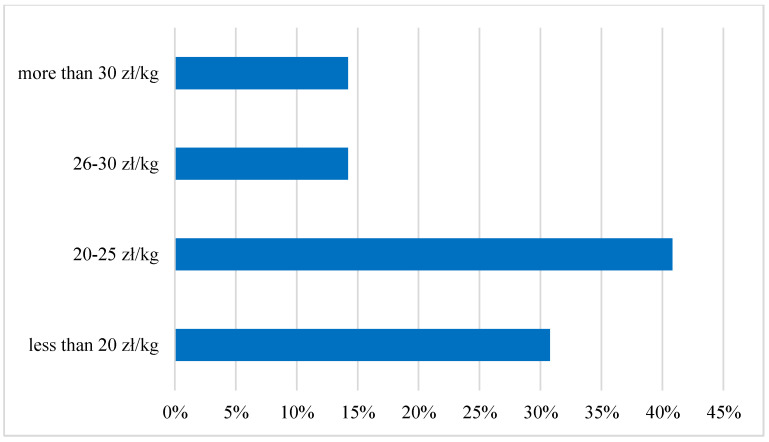
Price preferences for the boneless fillet of carp among the Young Adult Populations. Source: own elaboration based on data of [43].

In the case of a kilogram of fish fillet in the range from PLN 26 to PLN 30 and above PLN 30/kg, both variants gained 14% of the respondents’ votes. As a result, it turns out that only 14% of the respondents could afford to buy carp in the form of a boneless fillet, considering that the price per kilogram of fish in this form falls within this price range, as shown in Figure 6. It should be added that the price for 1 kg of fillet salmon is at least twice as much.

**Figure 7:**
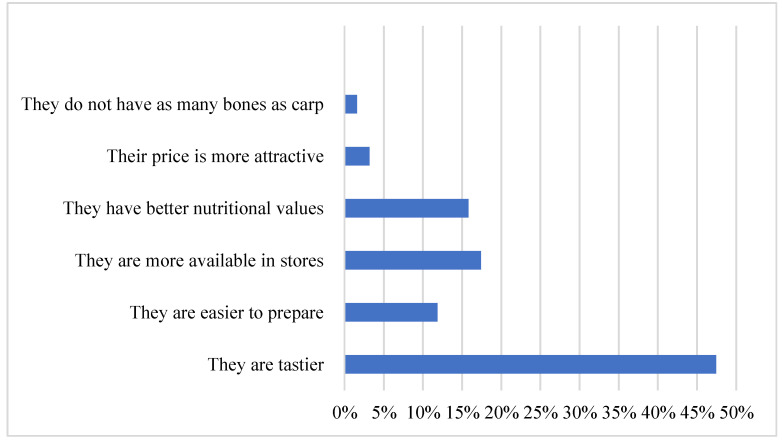
Preferences for sea fish among the Young Adult Populations in relation to carp. Source: own elaboration based on data of [43].

Another question in the survey was to explain why the respondents prefer sea fish more than carp. Among the proposed marine fish, several examples of the most popular species have been mentioned, including salmon, cod, flounder, salt and turbot. The results of the research have shown that as many as 47% of answers indicate that the sea fish mentioned in the question are perceived as tastier than the carp itself and that sea fish are more available in stores (17%). According to 16% of people filling in the questionnaire, the proposed sea fish has better nutritional values than carp, while according to 12%, it is easier to prepare, as shown in Figure 7.

**Figure 8:**
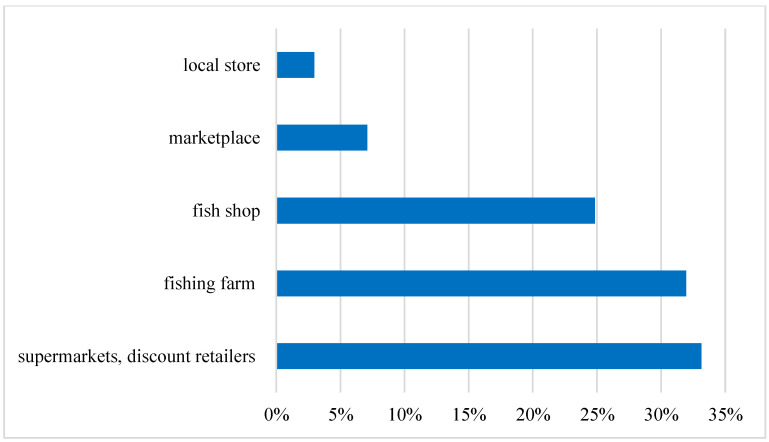
Shopping location preferences for carp among the Young Adult Populations in relation to carp. Source: own elaboration based on data of [43].

The next issue in the survey was the place where consumers prefer to buy carp. The answers to this question may explain their relation to both the quality and the price of the purchased fish, which may vary depending on the place of purchase. Hypermarkets, supermarkets, discount retailers (33%) and fishing farms (32%) turned out to be the places preferred by most of the surveyed consumers to buy carp. About 25% of respondents to the questionnaire buy supplies from a fish shop. Carp at the marketplace are purchased by 7% of respondents, and the remaining 3% choose the local store, as shown in Figure 8.

**Figure 9:**
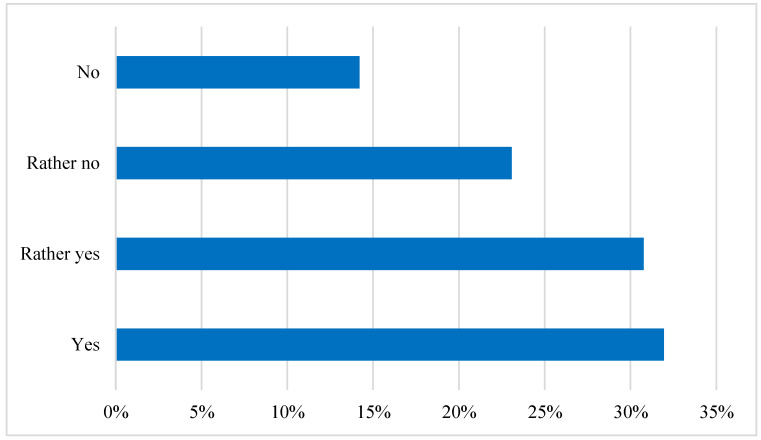
The importance of the origin of the purchased carp. Source: own elaboration based on data of [43].

The questionnaire also included a question about the importance attached to the origin of the purchased carp. For the majority of respondents, the source of the carp is important: ‘yes’ 32%, ‘rather yes’ 31%. In total, as many as 63% of respondents usually pay attention to where the fish they chose to buy was previously bred, as shown in Figure 9.

**Figure 10:**
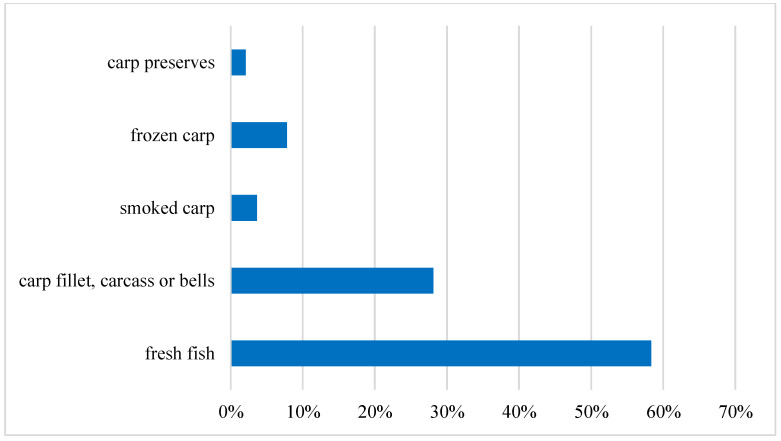
The preferences for the form of the purchased carp. Source: own elaboration based on data of [43].

When it comes to the form in which young consumers buy carp the most, nearly 60% declared that the most popular type of carp is fresh fish, most often found as a whole carcass with a head and scales, which must be trimmed and self-prepared. About 28% of the respondents choose a carp fillet, carcass or bells, which are much easier to prepare, but usually belong to the more expensive forms in which it is sold. The least popular are smoked carp, 4%, and carp preserves, 2%, which are, however, extremely rarely available on the market, as shown in Figure 10.

## 6. Discussion

The research has proved that in order to encourage young consumers to eat fish more often, including carp, the low-intensity aquaculture sector should build a solid carp market as soon as possible that meets the expectations of young consumers and matches current nutritional trends. However, this requires a fresh look at the issue of selling fish, which will be based on the commercial, promotion and marketing offers extended with new products. Carp has enormous market potential, which, however, requires the introduction of innovative products. This thesis is confirmed by the research by [44,45], according to which consumers pay more and more attention to farming and breeding methods and expect documented transparency of catches. Poles, and especially the younger generations, are becoming more and more aware of the negative practices of selling carp. Most of all, the promotion of the elimination of live carp sales, a humane way of killing them and the origin certification of fish from domestic farms are of increasing importance. Customers demand a product that will go to the most popular discount retailers in an easy-to-prepare form and convenient packaging. This thesis is supplemented by studies conducted by [21], which focused on the approach of Polish and German consumers to carp and the demand for new, innovative products from this fish. For this purpose, the surveyed consumers were asked to express their attitude to the new carp products. Research has shown that German consumers focus not only on the taste and ease of preparation but also on the convenience that comes with the appropriate grammage of the product. Among the preferred products were boneless carp fillet, vinegar carp balls, carp chips and carp ham. Polish consumers expressed a positive opinion mainly about boneless carp fillet. They decided that it would be the tastiest, the best for health and also easy to prepare, but they would also like to try carp ham or carp balls in vinegar. Research by [46] has confirmed that carp has a chance to be recognized on the Polish market. In their opinion, ‘the circle of potential carp consumers willing to buy and eat carp products more than once a year is very large and there is a possibility of increasing market demand for carp and carp preserves, provided that the product offer is adjusted to the needs and expectations of consumers’.

A positive example of activities promoting carp is conducted by the Association Partnership for the Barycz Valley, which for years has been focusing on achieving the sustainable development of tourism and expanding the market of local products and services based on specific natural and cultural conditions through [47]:

- Building the recognition of the Barycz Valley as a tourist area, especially good for active tourism surrounded by world-class nature;
- Using the promotion of tourism to endorse a sustainable fashion for local products and linking producers and service providers with the Barycz Valley;
- The use of world-class nature in building a brand that positively integrates the local community and creates a separate tourist model of the Barycz Valley.

Thanks to the Association’s initiative, Carp Days have been organized from September to November since 2005. In 2021, during the 16th edition of Carp Days, over 97 events (gastronomic, fishing, educational and outdoor) were organized, in which 32 entities (economic, social and public) were involved. A statistical participant of the Carp Days in the Barycz Valley was 31–45 years old, had higher education and was a resident of villages and large cities with over 100,000 inhabitants [48]. As the research has shown, product tasting is a very effective marketing tool supporting consumers’ interest in innovative products and increasing sales [49]. Highlighting local origin corresponds to consumer preferences for locally produced food [50] and can therefore support the demand for carp products, showing that carp also fits in with modern cuisine.

The findings of this study have to be seen in light of some limitations. First of all, they were conducted on a relatively small fraction of the Young Adult Populations. Secondly, they were carried out only in one area. However, due to the fact that the Barycz Valley is the biggest carp breeding center in Poland, and students from Wrocław University of Environmental and Life Study are in its proximity and have at least basic knowledge of sustainable development, it can be concluded that the research is representative for a wider population of young generation.

## 7. Conclusions

Based on the analysis, it can be concluded that changes on the demand side can be noticed in the agri-food products market. Increasingly noticeable is the emphasis on a healthy lifestyle combined with healthy, ecological food and outdoor activities in valuable natural areas combined with self-realization and passion. The aesthetics of the consumption area and the locality of production, as well as the convenience and comfort of transactions, are also gaining new value. However, this does not go hand in hand with the consumption of carp in Poland, which for most young consumers is associated mainly with a traditional Christmas dish and not an organic, healthy meal.

Even if the carp farming contributes to sustainable development, the consumption of the younger generation is definitely dominated by imported salmon, despite its higher price.

Young Adult Populations negatively relate to the carp fish bones and the smell, which is questionable, because of the past stereotypes that the carp ponds are slimy and muddy. They prefer sea fish because of the taste and availability of the stores, but this is due to the fact that there is simply no possibility to consume carp more frequently. Carp is available commonly in supermarkets and discout retailers only in the first half December. At other times, consumers have to go directly to the carp producer. The short supply chain in the carp market is still insufficiently developed, even if the origins of the production and the carp freshness are very important for Young Adult Populations.

On the basis of conducted analysis, it can be concluded that the situation on the carp market in Poland is not determined by the price level, but rather by factors beyond the price ones related to consumer tastes, or more broadly by sociological and psychological factors. This means that the development of the carp market in Poland requires diversification of the offer and marketing activities that influence the fashion and consumer preferences. Information activities are also necessary to highlight the health-promoting properties of carp meat.

The carp industry has great potential to create regional products, which is due to, among others, the rich national heritage, traditional carp breeding methods or the specific values of the natural environment. On the other hand, the main barrier to the development of these products is the relatively low knowledge about the possibility of selling them in other forms than before or the lack of financial resources dedicated to the development of marketing. A large number of regional products (including carp products) are not placed on the market, due to the fact that their production takes place occasionally, during local festivals and markets or is produced only for their own needs. Strengthening the marketing potential and increasing the frequency of visitors to carp regions is crucial for attracting potential customers, especially young, active consumers looking for an additional culinary and aesthetic experience. Therefore, without radical changes, it will not be possible to save the tradition of Polish carp fishing and at the same time protect valuable natural values.

## Figures and Tables

**Table 1:** Total aquaculture production by major producers in 2017.

Country	Value [in Thousands of EUR]	% Value	Volume [in Tonnes]	% Volume
China	131,860,769	59.56	64,358,481	57.48
Indonesia	11,424,415	5.16	15,896,100	14.20
India	10,882,498	4.92	6,182,000	5.52
Chile	9,216,766	4.16	1,219,747	1.09
Vietnam	8,599,847	3.88	3,831,241	3.42
Norway	6,954,930	3.14	1,308,634	1.17
Bangladesh	5,227,379	2.36	2,333,352	2.08
UE-28	5,059,021	2.29	1,372,012	1.23
Japan	4,147,649	1.87	1,021,580	0.91
South Korea	3,037,683	1.37	2,306,280	2.06
Thailand	2,393,042	1.08	889,891	0.79
OTHERS	22,591,846	10.2	11,246,153	10.05
In total	221,395,844	100.00%	111,965,471	100.00%

Source: own elaboration based on [7].

**Table 2:** Amount of feed and water necessary to produce 1 kg of meat.

	Feed [in kg]	Water [in Litres]
Beef	8	14,500
Pork	3	5990
Poultry	2	4330
Salmon	1.1–1.2	1500

Source: own elaboration based on [9,10].

**Table 3:** Top 10 aquaculture species in the world.

	Aquaculture Species	Production in Tonnes	%	Value in 10^3^ USD	%
1.	Grass carp	5,822,869	7.60	7,462,316	4.73
2.	Silver carp	5,125,461	6.69	6,776,963	4.29
3.	Common carp	4,328,083	5.65	5,905,279	3.74
4.	Japanese carpet shell	4,049,541	5.29	3,708,929	2.35
5.	Nile tilapia	3,930,579	5.13	6,017,377	3.81
6.	Whiteleg shrimp	3,879,786	5.07	18,899,320	11.97
7.	Bighead carp	3,402,870	4.42	4,373,102	2.77
8.	Catla	2,764,944	3.61	4,813,647	3.05
9.	Atlantic salmon	2,381,576	3.11	11,945,146	7.56
10.	Rohu	1,785,900	2.33	3,034,446	1.92

Source: [6].

**Table 4:** Production of common carp in the UE in 2018.

Country	Production in 2018 (in Tons)	% of Change 2018/2008
Poland	20,751	+21%
Czech Republic	18,430	+5.2%
Hungary	11,462	+9.3%
Germany	4746	−43.7%
France	Estimated: 3000–4000	Estimated: −20%

Source: [15].

**Table 5:** Fish farms in the Barycz Valley.

Number of Fish Fams	The Total Area of the Ponds [in ha]	Production Value Measured in Income from Fishing Activities Together with the Total Value of Production [in PLN]	Number of People Employed on a Permanent Basis in Fish Farms
26	8253.45	25,348,966.56	271

Source: own elaboration based on [38].

**Table 6:** Diversification of the fish farms activities.

Activities	
Year-round sale of live fish Gastronomy Processing—pre-treatment Processing—advanced treatment Growing cereals Nature education	Sale of stocking material Agricultural retail trade Agritourism Live fish sale before Christmas Cyclical sale (e.g., gastro-zone) Services for anglers

Source: own study based on the conducted research.

## Data Availability

The data presented in this study are available on request from the corresponding author. Part of the research data come from the Master thesis of Aleksandra Kondela “Prospects for the development of aquaculture in Poland” under supervision of dr. Magdalena Raftowicz, Wrocław University of the Environmental and Life Sciences.

## Appendix A

- How often do you eat a fish meal?
- How often do you eat any of the listed species of fish?
- How often do you eat a meal that includes carp (any form?)
- What are the factors that encourage your carp consumption?
- Identify factors that discourage carp consumption
- If you were in a restaurant, would you decide to eat a carp dish?
- Would easier and year-round access to carp encourage you to consume this fish more often
- Could a relatively low and affordable price mean that carp would appear on your table more often?
- What price are you willing/willing to pay per kilogram of fish as fillet?
- Why do you prefer sea fish such as salmon, cod, flounder, sole, turbot, rather than carp?
- Identify the place where you most often buy carp
- Do you pay attention to the origin of the carp you buy?
- What form do you purchase carp in?
